# Choriocarcinoma Syndrome: A Potentially Fatal Complication of Testicular Cancer

**DOI:** 10.1155/2019/4092941

**Published:** 2019-02-24

**Authors:** Vicken Sarkis Zeitjian, Waqas Arslan, Andres Borja-Alvarez, Surabhi Amar

**Affiliations:** ^1^Creighton University Arizona Health Education Alliance, Department of Internal Medicine, USA; ^2^Creighton University Arizona Health Education Alliance, Department of Hematology and Oncology, USA; ^3^Creighton University Arizona Health Education Alliance, Department of Pulmonary and Critical Care, USA

## Abstract

Choriocarcinoma syndrome is a rare clinical entity with advanced, high volume choriocarcinomatous tumors and markedly elevated B-hCG (>50,000 IU/L). Recognition is important because the diagnosis of this syndrome identifies poor prognosis without mortality-proven management options. We present a case of a male in his twenties with metastatic choriocarcinoma who developed choriocarcinoma syndrome acutely after chemotherapy commencement. The patient deceased after hypoxic respiratory failure due to diffuse alveolar hemorrhage as a result of death of vascular tumors. While the prognosis for early diagnosis and treatment is excellent, the prognosis for late diagnosis is grim. Unfortunately, despite surgical or chemotherapeutic intervention, this syndrome has poor outcome.

## 1. Introduction

Testicular neoplasms are the most common solid malignancy in males between the ages of 15 and 35 years old. Germ cell tumors (GCTs) account for ~95% of testicular neoplasms. Of those, choriocarcinoma is the least common (1%), yet most aggressive type. Choriocarcinoma often presents as an element of the mixed GCTs; however, pure choriocarcinoma, as in our patient, is rare [1]. Choriocarcinoma is characterized by the production of human chorionic gonadotropin (hCG) molecule, and levels of >50,000 IU/L often signify choriocarcinoma syndrome, which is associated with a poor prognosis [2].

## 2. Case

**Figure 1:**
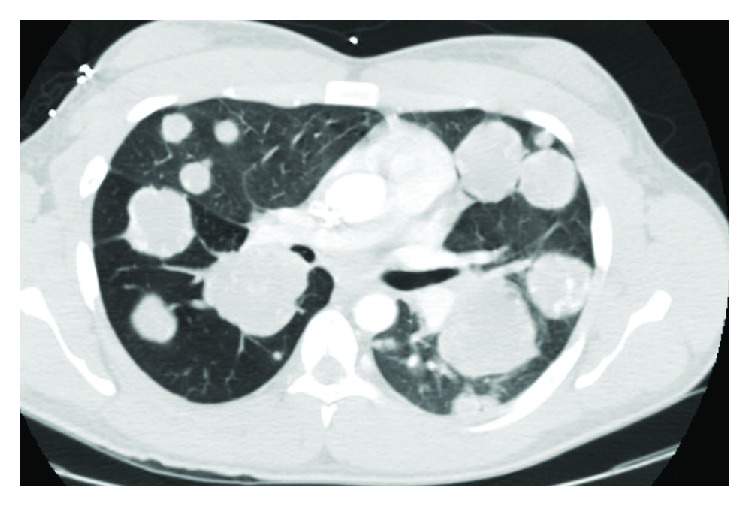
CT chest with pulmonary metastatic disease in a “cannonball” appearance.

A healthy 22-year-old male presented with a two-week history of hemoptysis, weight loss of 10 pounds, generalized fatigue, and persistent night sweats. On physical examination, he had scattered rhonchi in both lungs. There was no palpable peripheral lymphadenopathy or organomegaly in the abdomen. The testes were normal to exam without palpable masses. The CT of the chest revealed innumerable pulmonary nodules concerning for metastatic disease (Figure 1). CT abdomen showed a 6.9 × 8.0 × 7.6 cm retroperitoneal mass. An US of the testicles was done showing an ill-defined hypoechoic structure measuring 8 mm within the right testicle. He underwent a CT-guided lung nodule biopsy, which revealed metastatic choriocarcinoma. The serum B-hCG level was 274,465 IU/L. The patient underwent right orchiectomy consistent with choriocarcinoma. A diagnosis of stage IIIC choriocarcinoma of the right testis with metastasis to the retroperitoneum and lungs was made. He delayed the treatment to seek another opinion and elected to proceed with sperm banking. 16 days after diagnosis, the patient returned to the emergency department with worsened hemoptysis, melena, tachycardia, tachypnea, and hypoxia. His serum B-hCG level had increased to 1,629,195 IU/L. The patient was transferred to the medical intensive care unit, and chemotherapy was started on an urgent basis with etoposide, ifosfamide, and cisplatin (VIP) with mesna support.

Over the next few days, his respiratory status declined as he developed severe acute respiratory distress syndrome and, therefore, was intubated. Due to the high tumor burden and worsening of his clinical status after the initiation of chemotherapy, a diagnosis of choriocarcinoma syndrome was made. He completed 5 days of planned chemotherapy but remained in critical condition needing increased ventilator support. In spite of maximal respiratory support, the patient remained hypoxic. Extracorporeal membrane oxygenation was considered, but due to gastrointestinal bleeding, he was not a candidate. He eventually passed away due to hypoxic cardiac arrest.

## 3. Discussion

Many clinicians may be unaware of the entity “choriocarcinoma syndrome” due to the rarity of pure testicular choriocarcinomas. The first case was described in 1984, and since then, there are a handful of cases describing this entity. Logothetis defined choriocarcinoma syndrome as a distinct clinical presentation of advanced germ cell tumors with high volume choriocarcinomatous elements and a markedly elevated B-hCG. Elements include hemorrhagic sequela of metastatic disease in any organ system involved [3]. Choriocarcinomas are heavily vascular; therefore, they often present with life threatening hemorrhage from metastatic sites [4]. Lung metastasis leading to diffuse alveolar hemorrhage is the most common manifestation as seen in our patient; however, literature describes bleeding at other metastatic sites including the liver, kidneys, bone, small intestines, and brain. Death usually results from asphyxia due to alveoli flooding with blood [5].

The syndrome is reported to occur in 2 clinical settings: spontaneously in advanced disease or more commonly acutely after chemotherapy. Oftentimes, dry cough or hemoptysis due to metastatic lung disease is the first presenting symptom; at that point, staging and identifying tumor burden is important for prognosis [6]. The serum B-hCG level can be used as a marker of disease progression. Early recognition of choriocarcinoma and prompt treatment is essential as fatal lung bleeding may occur in the first few days of chemotherapy commencement. The Annals of Oncology suggests that avoiding full-dose chemotherapy during initial treatment may possibly reduce this complication; however, there are few data available on how to optimally administer early induction chemotherapy. In choriocarcinoma syndrome, Honecker et al. recommend 2–3 days of full-dose cisplatin and etoposide, with continuation of chemotherapy when the patient has recovered (e.g., day 14) [7]. Unfortunately, diffusely metastatic disease, despite surgical or chemotherapeutic intervention, has proven to have poor outcome, and optimal management of advanced disease remains difficult.
